# The Effect of Different Scaling Methods on the Surface Roughness of 3D-Printed Crowns: An In Vitro Study

**DOI:** 10.3390/ma18245525

**Published:** 2025-12-09

**Authors:** Turki Alshehri, Dhai Albishri, Aminah M. Alsayoud, Abdulkarim Alanazi, Faisal Masaud, Anas A. Howsawi, Abdulrahman A. Balhaddad

**Affiliations:** 1 Department of Substitutive Dental Sciences, College of Dentistry, Imam Abdulrahman Bin Faisal University, P.O. Box 1982, Dammam 31441, Saudi Arabia; taalshehri@iau.edu.sa; 2 College of Dentistry, Imam Abdulrahman Bin Faisal University, P.O. Box 1982, Dammam 31441, Saudi Arabiakalanazi98@gmail.com (A.A.); faisal.s.masaud@gmail.com (F.M.); 3 Dental Hospital, College of Dentistry, Imam Abdulrahman Bin Faisal University, Dammam 34212, Saudi Arabia; 4 Department of Restorative Dental Sciences, College of Dentistry, Imam Abdulrahman Bin Faisal University, P.O. Box 1982, Dammam 31441, Saudi Arabia

**Keywords:** 3D-printing, crown, roughness, scaling, resin

## Abstract

Despite the growing adoption of 3D-printed resin materials, limited evidence exists on how clinical procedures such as scaling impact their surface integrity. This in vitro study evaluated the effect of different scaling methods on the roughness of five 3D-printed resin materials. Disc-shaped specimens from five commercially available 3D-printed crown materials (Crowntec, Varseosmile, Freeprint, Ezprint, and C&B Nextdent) were fabricated. Each material group (N = 30) was subjected to three scaling techniques: stainless-steel manual, plastic, and piezoelectric ultra-sonic scalers. Surface roughness (Ra) was quantified using a non-contact profilometer before and after scaling. One-way ANOVA was used to analyze the data. All the crowns exhibited increased roughness when treated with ultra-sonic scalers compared to the other scalers, and this was significant (*p* < 0.05) for all except VarseoSmile. Compared to the other crowns, Freeprint crowns (0.07 ± 0.04 µm) showed the greatest roughness change with plastic scalers, which was significant (*p* < 0.05) compared to C&B Nextdent crowns (0.001 ± 0.03 µm). For hand and ultra-sonic scalers, no significant differences were found among the crown types (*p* > 0.05). In conclusion, ultra-sonic scalers, while efficient, may increase surface roughness, potentially enhance plaque retention, and compromise prosthesis longevity.

## 1. Introduction

In recent years, 3D printing has revolutionized dentistry, offering promising materials that relies heavily on digital fabrication methods [1,2]. Despite being around for decades, subtractive computer-aided design/computer-aided manufacturing (CAD/CAM) methods are currently the most often utilized digital manufacturing variations in dentistry [3]. Over the past several years, subtractive manufacturing (SM) like milling and grinding have been the primary means of fabricating dental restorations; nevertheless, these processes have several drawbacks, including material waste, reliance on the milling instrument’s geometry, and lengthy processing times [4,5].

Additive manufacturing (AM) represents an alternative approach for generating CAD/CAM models, where the final object is built layer by layer using three-dimensional (3D) digital data [6,7]. This 3D-printing technique offers several advantages, such as reduced material consumption, lower heat and noise generation, and the ability to fabricate complex geometries simultaneously, thereby decreasing both production time and cost [6,7]. Various 3D-printing technologies exist, including stereolithography (SLA) and digital light processing (DLP). Among these, liquid crystal display (LCD) printers—a subtype of SLA—are commonly used in dental clinics due to their cost-effectiveness and ease of operation [8].

There are several applications of 3D printing in prosthodontics and restorative dentistry. Among them, the fabrication of permanent and temporary indirect full and partial coverage restorations has gained popularity due to the reduced treatment time. SLA is a vat-polymerization process where a laser selectively cures the resin by following a specific pattern on its surface. It is recognized for its superior accuracy, fine detail reproduction, and high resolution, outperforming conventional methods used for indirect restorations [9]. On the other hand, DLP employs a digital light projector to cure resin layer by layer through photopolymerization. It is especially appreciated for its faster printing speed, addressing the extended production time typically associated with SLA [9,10].

One of the most important criteria for evaluating the success of definitive restorations is their long-term durability. This is largely influenced by the patient’s oral hygiene and the mechanical performance of the materials used as a final prosthesis [11]. Similar to natural teeth, dental crowns are subjected to plaque accumulation, and bacterial colonization, especially at the tooth–crown interface [12,13]. Plaque and calculus accumulations can be eliminated from dental crowns via regular cleaning visits. Consistent removal of these deposits as a preventive measure can prevent the onset of gingivitis and periodontists [14].

Regular dental cleaning can be achieved by scaling and root planing, playing a vital role in maintaining periodontal health by eliminating biofilm and calculus, as well as performing pocket debridement [14]. Professional oral hygiene procedures utilize various curettes and scalers, including universal and specialized types, along with piezoelectric ultra-sonic scalers with different types of scaling tips such as plastic, stainless steel, or titanium [15,16]. Moreover, the material of scaling tips serves specific purposes; for instance, plastic tips are specifically recommended for implant-supported restorations to prevent damage that could compromise implant longevity and osseointegration [17]. However, it was found that ultra-sonic and hand scalers may cause scratches and increased roughness when cleaning enamel [18,19]. Therefore, these scalers may also have the potential to alter the surface roughness of dental restorations, resulting in undesirable outcomes such as plaque accumulation and color change [16].

Previous studies reported that scaling methods can be a factor in increasing the surface roughness of the final restoration materials [20,21]. A study by Ellakany et al. evaluated the effect of three different scaling methods, a hand scaler, plastic scaler, and ultra-sonic scaler, on the surface of different CAD/CAM ceramic materials. A significant increase in surface roughness was observed among all ceramic groups, with the highest roughness values associated with the ultra-sonic scaler followed by the manual and plastic scalers [16]. It is worth mentioning that no study has yet evaluated the impact of different scaling methods on 3D-printed resin permanent crowns. Therefore, this study evaluated the change in surface roughness among five 3D-printed resin permanent crowns when subjected to ultra-sonic, hand, and plastic scalers. The null hypothesis of the study is that there would be no significant differences in surface roughness change between the investigated scaler types or the 3D-printed crowns.

## 2. Materials and Methods

### 2.1. Data Setting, Study Group, and Sample Size Calculation

The current study used three different scaling methods to assess the effect of scaling methods on the roughness of five 3D-printed resin materials (Table 1). Following the same materials as in a previous study [16], the scaling methods are described below.

(1) Plastic scaler: a plastic tip composed of Plasteel, a high-grade unfilled resin (Implacare II LG1/2, Hu-Friedy Mfg. Co., Chicago, IL, USA).
(2) Hand scaler: a stainless-steel metal tip (H3/H4 Jacquette Scaler, Hu-Friedy Mfg. Co., Chicago, IL, USA).
(3) Ultra-sonic scaler: a piezoelectric scaler tip (Piezo Scaler Tip 201, KaVo PiezoLED Ultraschall Scaler, Kaltenbach & Voigt GmbH, Biberach, Germany).

**Figure 1:**
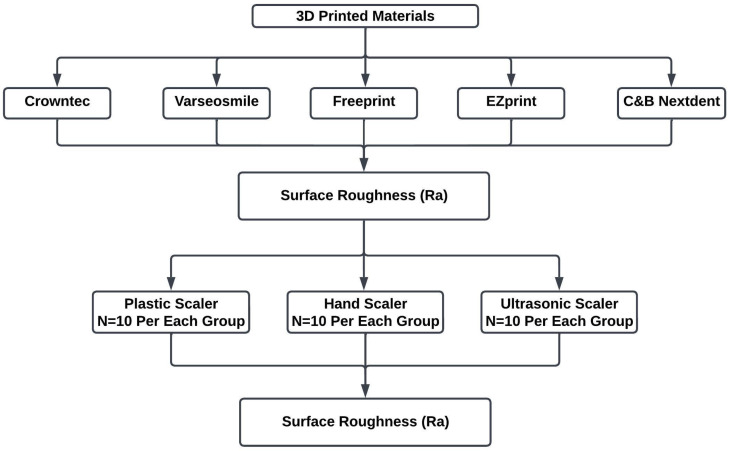
Sample distribution and assessment of the 3D-printed resin materials.

Each 3D-printed resin was subjected to the three scaling methods (N = 10 per scaling method; N = 30 per material), resulting in a total of 150 samples and 15 groups as shown in Figure 1. For the sample size calculation, a priori sample size calculation was carried out using G*Power 3 to establish the sample size necessary for one-way ANOVA. The significance level was set at *p* < 0.05. The results indicated a required total sample size of nine samples per group to detect large effect sizes (f = 0.4). Power was set at 80%. In addition, previous studies indicate the change in surface roughness mainly required five to ten samples per group to detect differences between the groups [16,22,23]. Therefore, in this study, each group consisted of ten samples.

### 2.2. Sample Preparation

Disc-shape specimens were designed virtually with 10 mm diameter and 2 mm thickness. Then, the design was exported as standard tessellation language (STL) files into a compatible format for the 3D-printing system. A total of five 3D-printed temporary and permanent discs were used in the current study (Table 1). All specimens were printed using the following parameters: 50 µm layer thickness with 0° printing orientation, unpolymerized acrylic resin removed using isopropyl alcohol 99.9% liquid (Saudi Pharmaceutical Industries, Riyadh, KSA). The post-curing process was performed for all of the samples for 15 min per surface, resulting in a total post-curing time of 30 min [24,25]. Printed support structures were removed using 800, 1500, and 2000 grit sizes of silicon carbide paper, followed by water rinsing as previously suggested [26].

### 2.3. Surface Roughness Assessment

After 3D printing, the specimens were subjected to a finishing and polishing procedure via different grit sandpapers mounted on the polishing machine disc (AutoMet250, BUEHLER, Lake Bluff, IL, USA). Polishing was conducted for up to #6000 grit sandpaper [27]. Then, the average surface roughness (Sa) of the printed resin specimens was measured using a non-contact optical profilometer (Contour Gt-K 3D optical profiler, Bruker Nano GmbH, Berlin, Germany). Using a prefabricated mold that guided the scan of various regions at the center of the specimens, three random readings were taken at the speed of 0.5 mm per second, with a cutoff of 0.8 mm, scan area of 639 μm × 479 μm, lateral resolution of 0.33 μm, vertical resolution of <0.1 nm, and magnification of ×20. The average for the three readings was calculated [28].

### 2.4. Scaling Methods and Calculating the Change in the Average Surface Roughness

Forty strokes of manual scaling, for both the plastic and hand scalers, were applied to the surface of the 3D-printed resin with a 75° working angle (for approximately 40 s) and moderate lateral pressure [6,16]. Using water irrigation at a flowrate of 13 mL/min, an ultra-sonic scaler was used at a 0-degree angle [12,29] with an approximate standard lateral force of 0.2 N, frequency of 28–36 kHz, and stroke amplitude of 40 μm [30]. All of the parameters used were applied following the manufacturer’s recommendations (KaVo PiezoLED Ultrasonic Scaler, Kaltenbach & Voigt GmbH, Biberach, Germany). Scaling was carried out from the bottom of the sample, passing the center, and moving to the top of the sample [12]. The surface was subjected to 40 strokes with a stroke length of 8 mm. The borders of the travel path were marked to be aligned with the prefabricated mold. Renewing the curettes and piezoelectric tips was after performed after every five specimens, and the specimens were cleaned after scaling using water path sonication with distilled water. After scaling, the average surface roughness was measured again as described above. The change in the average surface roughness was measured by subtracting the baseline values from the post-scaling values.

### 2.5. Statistical Analysis

Statistical analysis was performed using Sigma Plot 12.0 (SYSTAT, Chicago, IL, USA). Descriptive statistics, including the mean, standard deviation, frequency, and percentages, were used to summarize the change in average surface roughness. A one-way ANOVA with Tukey’s post hoc test was conducted to compare the change in surface roughness among the three scaling methods and the five 3D-printed resin materials. A paired *t*-test was used to compare pre- and post-scaling values. To ensure the validity of the parametric tests, normality was assessed using the Shapiro–Wilk test. If the assumptions of normality were violated, non-parametric Kruskal–Wallis and Wilcoxon signed rank tests were performed instead. A 5% significance level (*p* < 0.05) was used for all statistical tests to determine statistical significance.

## 3. Results

**Figure 2:**
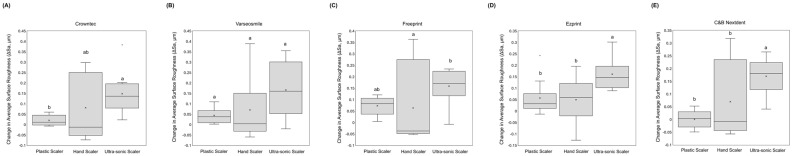
The impact of the different scaling methods on the average surface roughness change (ΔSa, µm) of the 3D-printed crowns: (**A**) Crowntec, (**B**) Varseosmile, (**C**) Freeprint, (**D**) Ezprint, and (**E**) C&B Nextdent. Different letters indicate significant differences (*p* < 0.05) among the scaling methods.

Figure 2 illustrates the effects of plastic, hand, and ultra-sonic scalers on the roughness of 3D-printed discs. In Figure 2A, Crowntec discs exhibited a significant increase in roughness (*p* < 0.05) when treated with ultra-sonic scalers (0.15 ± 0.10 µm) compared to plastic scalers (0.02 ± 0.02 µm). Figure 2B shows that there were no significant differences in roughness among the scaler types for VarseoSmile discs, although ultra-sonic scalers resulted in the highest roughness (0.17 ± 0.14 µm). In the Freeprint discs (Figure 2C), ultra-sonic scalers (0.16 ± 0.07 µm) produced significantly greater roughness (*p* < 0.05) than hand scalers (0.06 ± 0.17 µm). Figure 2D indicates that Ezprint discs also exhibited higher roughness with ultra-sonic scalers (0.16 ± 0.06 µm), which was significantly greater (*p* < 0.05) than the roughness observed with plastic (0.06 ± 0.07 µm) and hand scalers (0.05 ± 0.10 µm). Lastly, Figure 2E demonstrates that ultra-sonic scalers (0.17 ± 0.07 µm) caused a significant increase in roughness (*p* < 0.05) compared to both plastic (0.001 ± 0.03 µm) and hand scalers (0.07 ± 0.15 µm).

**Figure 3:**
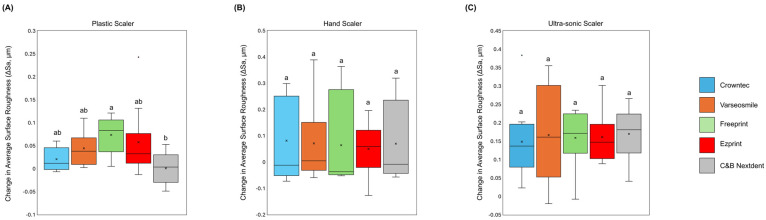
A comparison of the average surface roughness change (ΔSa, µm) of the 3D-printed crowns following each scaling method: (**A**) plastic scaler, (**B**) hand scaler, and (**C**) ultra-sonic scaler. Different letters indicate significant differences (*p* < 0.05) among the scaling methods.

**Figure 4:**
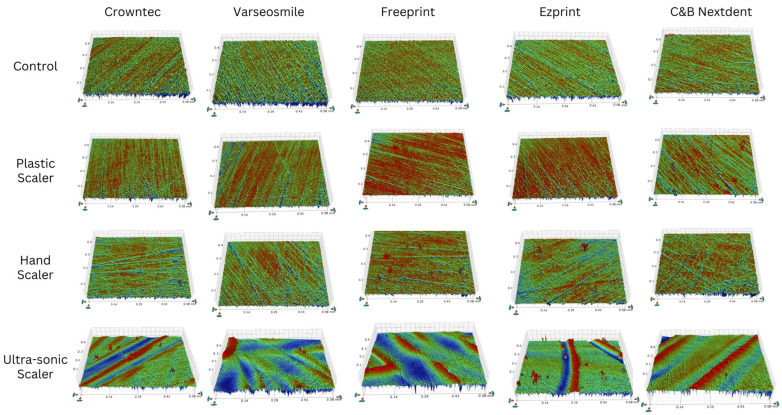
Representative scans for surface roughness change for the investigated 3D-printed crowns and scaling techniques. The green color indicates minor changes in roughness, blue represents moderate changes, and red signifies high roughness changes.

Figure 3 compares the roughness variations among different types of 3D-printed crowns for each scaler. In Figure 3A, Freeprint crowns (0.07 ± 0.04 µm) showed the greatest roughness change with plastic scalers, which was significant (*p* < 0.05) compared to C&B Nextdent crowns (0.001 ± 0.03 µm). For hand and ultra-sonic scalers (Figure 3B,C), no significant differences were found among the crown types (*p* > 0.05). Figure 4 presents the roughness scans for all groups, highlighting that the ultra-sonic scaler generally resulted in greater roughness changes compared to plastic and hand scalers.

Table 2 illustrates the baseline and post-scaling values where a comparison using a *t*-test was performed. In plastic and ultra-sonic scalers, a significant increase (*p* < 0.05) was observed in all the groups, except C&B Nextdent following the plastic scaler. In the hand scaler groups, the normality test failed and Wilcoxon signed rank test was used. No significant difference was observed between pre- and post-scaling values.

## 4. Discussion

The present study was conducted to assess the impact of various scaling techniques using different scaler tip materials on the surface properties of five different 3D-printed permanent and temporary crown resins (CT, FP, EZ, VS, and ND). The findings indicate that all the 3D-printed crowns exhibited a significant increase in surface roughness when treated with ultra-sonic scalers compared to plastic and hand scalers. When the 3D-printed crowns were compared, there was a significant difference between them only when the plastic scaler was used. Based on these findings, the null hypothesis of the present study was partially rejected. The results of the study are expected to assist dental practitioners in selecting the most appropriate instrumentation when treating patients with 3D-printed resin materials, to preserve the surface smoothness of definitive and long-term temporary restorations. In addition, the brand selected when 3D-printing crowns does not seem critical, except if a plastic scaler will be used.

Multiple studies have evaluated the effects of scaling on various restorative materials such as ceramics [16], glass ionomer restoration [31], and flowable composite [32]. However, there is a notable lack of research in addressing the relation between 3D-printed permanent and temporary crown materials and scaling techniques. The present study is therefore the first to address this gap, providing baseline data for future investigations. In terms of study materials, lower resistance to ultra-sonic scaling was observed in the Crowntec permanent resin among the different scaler tip groups, whereas the Varseosmile material demonstrated acceptable resistance with no significant differences across all scaling methods. A previous study comparing the surface hardness of Varseosmile and Crowntec reported no significant differences between the two materials, although Varseosmile showed slightly higher microhardness values than Crowntec [24]. These results highlight the differences between temporary resin (Crowntec) and permanent resin (Varseosmile), which may explain the inherent properties of the Varseosmile material and its relatively greater resistance to the external forces applied during various scaling methods.

The changes in surface roughness of 3D-printed restorations observed with various scaler types may be attributed to differences in the scaler tip material—plastic or metal—and the magnitude of applied force, as previously discussed [16,33]. In the present study, ultra-sonic scaling was applied as a simulation of professional surface debridement, which is among the most frequently performed supportive procedures following the definitive cementation of prosthetic restorations [34]. Ultra-sonic instrumentation consistently resulted in the highest surface roughness values across nearly all resin groups, with statistically significant increases observed in several crown types. This finding is consistent with previous studies reporting the high abrasiveness of ultra-sonic scalers on resin-based restorative materials [12,35]. The combined effect of vibrational energy, water spray, and direct contact with a metallic tip can compromise the resin matrix, expose filler particles, and create irregularities. This increase in roughness is likely due to the mechanical linear vibration generated by the ultra-sonic tips. These forces act on the ceramic material, displacing and pulling grains from its matrix. As these grains are removed, the surface becomes less smooth and more textured, contributing to the higher roughness observed with the ultra-sonic scalers.

In this study, the plastic scaler produced a smoother surface compared to the ultra-sonic scaler. Similarly, Ellakany et al. [16] evaluated four different ceramic materials using various scaler tip types, and their findings showed that plastic scalers caused less surface deterioration than ultra-sonic tips. Additionally, another study reported that plastic scaler tips resulted in smoother surfaces on zirconia restorations [30]. On the other hand, variable results were observed when comparing plastic and manual scaler tips, where the plastic scaler produced a slight increase in the mean surface roughness in the Freeprint resin materials. Another study evaluated the effects of metal and plastic scaler tips on titanium surfaces and suggested that plastic scalers can alter surface properties when compared to metal scalers [36]. However, the materials used in that study differ significantly from 3D-printed resins, and the differences in study design and evaluation methods may account for the conflicting results. Moreover, the metal tip group exhibited a higher standard deviation and a greater number of elevated surface roughness values than the plastic scaler group, although no statistically significant differences were found between the two scaler types or material groups.

After evaluating the three different scaling methods, no significant differences were observed among the materials studied. The chemical structures of these materials vary in terms of the resin matrix composition, filler loading, and types of photoinitiators used. It is important to note that there is generally a lack of detailed information regarding the chemical composition of these materials, which may limit our ability to discuss the study’s findings in this context. Nonetheless, while the materials exhibited varying surface roughness values, the differences were not significant, except for the comparison between Freeprint and C&B Nextdent when a plastic scaler was used. In summary, it can be stated that the composition of the materials did not significantly influence the crowns’ responses to the scaling force, suggesting that any changes in roughness were primarily dictated by the type of scaler employed.

Increased surface roughness can negatively affect the mechanical and physical properties of dental restorations, particularly by promoting bacterial adhesion as rough surfaces serve as favorable sites for microbial colonization [23,37]. Previous studies have reported a clinically acceptable surface roughness (Sa) value of approximately 0.2 µm; values exceeding this threshold are associated with increased bacterial adhesion and biofilm formation [37]. Therefore, the scaling technique used on dental restorations should be carefully selected to minimize surface damage. A study by Checketts et al. [21] evaluated microbial adhesion on zirconia, lithium disilicate, and Type III gold alloys after different scaling procedures. The study found that lithium disilicate exhibited a significant increase in surface roughness following scaling compared to its untreated control, which led to higher bacterial adhesion of *Streptococcus mutans*, *Lactobacillus acidophilus*, and *Actinomyces viscosus*. In contrast, zirconia demonstrated lower surface roughness and reduced microbial adhesion. These results highlight the critical role of surface roughness induced by scaling in influencing bacterial colonization on restorative materials.

The primary focus of this study was to compare the roughness values of crowns following the use of different scaler types, making it essential to calculate the changes in surface roughness to effectively illustrate the results. However, it is important to note that the reported roughness values reflect the differences between baseline and post-scaling measurements, suggesting that the actual roughness is higher. Therefore, Table 2 was designed to illustrate the pre- and post-scaling values. At baseline, all ceramic materials exhibited a roughness value ranging from 0.14 to 0.22 µm. The post-scaling roughness values exceeded the critical threshold of 0.20 µm across all groups, except for Crowntec, Varseosmile, and C&B Nextdent when plastic scalers were used. These findings suggest that these brands could be prioritized when designing 3D-printed resin crowns and should be cleaned using plastic scalers. For other brands or different scaling methods, it is recommended to apply less force when cleaning crowns or to polish them afterward with intraoral polishing kits.

While increased surface roughness is associated with a higher risk of microbial attachment, its impact is not limited to biological concerns. Mechanically, elevated surface roughness has also been linked to a greater likelihood of surface cracks, which can contribute to brittleness and reduced structural integrity in resin-based restorative materials [38]. To address these concerns, surface finishing is recommended, as it has a significant effect on both color stability and surface smoothness in 3D-printed restorative materials [39]. Careful control of surface topography through appropriate post-printing finishing procedures is essential. These measures help to minimize stain susceptibility and enhance the esthetic longevity of the restoration.

The findings have direct clinical implications. While ultra-sonic scalers are highly effective in removing calculus, they also carry the risk of increasing surface roughness on resin-based and 3D-printed restorations. Such alterations can compromise both the aesthetic appearance and biological outcomes, particularly in anterior regions or in patients at higher risk of periodontal disease. Therefore, plastic or hand scalers may represent safer alternatives for routine maintenance of these restorations. Several limitations should be acknowledged in the current study. First, the tested samples were polished without the application of glaze materials, which may have contributed to variability in surface roughness values. Second, the force of manual scaling was applied by an operator. Using adopted instrumented rigs could be beneficial to standardize the applied force among the groups. Additionally, the study was limited to the available materials and did not include a comparison of all permanent and temporary 3D-printed crowns. Third, this study examined the effects of scaling methods without the influence of water or artificial aging. Investigating the impact of scaling after aging may provide deeper insights into the interaction between scaling and aging on the roughness of the materials. Finally, future research may investigate other surface properties, such as gloss, wear, hardness, bacterial adhesion, and surface visualization using scanning electron microscope (SEM) images. Additionally, the effects of scaling after repeated cycles should be considered.

## 5. Conclusions

In conclusion, this study introduces a new insight into the impact of various scaling techniques on the surface roughness of 3D-printed resin materials. The present study demonstrated that ultra-sonic scalers consistently produced the highest surface roughness values, whereas plastic and manual scalers generally caused fewer surface changes. Furthermore, the type of material does not appear to be a determining factor. This highlights the significance of selecting appropriate scaling methods to optimize dental treatment outcomes. Further assessments are required to investigate other non-tested variables.

## Figures and Tables

**Table 1:** The list and composition of the 3D-printed resin materials used in the study.

	Crowntec	Varseosmile	Freeprint	EZprint	C&B Nextdent
Manufacturer	Saremco Dental, Rebstein, Switzerland	BEGO Bremer Goldschlägerei Wilh. Herbst, Bremen, Germany	DETAX, Ettlingen, Germany	Aidite (Qinhuangdao) Technology (Qinhuangdao, China)	Nextdent (Soesterberg, The Netherlands)
Composition	4,4′-isopropylidenediphenol (ethoxylated) and 2-methylprop-2-enoic acid (i.e., methacrylic-acid-ester-based resin), silanized dental glass fillers, and pyrogenic silica (inorganic fillers ~30–50% by mass, particle size ~0.7 µm), plus photoinitiators/initiators	Methacrylate-based composite resin composed of esterification products of 4,4′-isopropylidenediphenol (ethoxylated) and 2-methylprop-2-enoic acid, with silanized dental glass fillers (≈30–50 wt.%, ~0.7 μm), and a photoinitiator system containing diphenyl(2,4,6-trimethylbenzoyl)phosphine oxide and methyl benzoylformate; minor pigments such as titanium dioxide and iron oxides are included	Methacrylate-based permanent crown resin composed primarily of an alkoxylated phenol derivative, methacrylate-terminated (≈40–<60 wt.%), combined with 7,7,9-trimethyl-4,13-dioxo-3,14-dioxa-5,12-diazahexadecane-1,16-diyl bismethacrylate (≈5–<20 wt.%), and 1,6-hexanediol dimethacrylate (≈5–<20 wt.%); minor monomeric components include hydroxypropyl methacrylate (≈0.1–<5 wt.%); the photoinitiation system consists of diphenyl(2,4,6-trimethylbenzoyl)phosphine oxide and phenyl-bis(2,4,6-trimethylbenzoyl)phosphine oxide (each ≈0.1–<5 wt.%)	Not available	Bisphenol-A ethoxylated dimethacrylate (Bis-EMA ≥50–<70 wt.%) and Triethylene glycol dimethacrylate (TEGDMA ≥10–<20 wt%), with Diphenyl(2,4,6-trimethylbenzoyl)phosphine oxide (TPO ≥0.1–<1 wt.%) as the photoinitiator
Layer thickness	50 µm	50 µm	50 µm	50 µm	50 µm
Printing technology	LED-based digital light processing (DLP)	LED-based digital light processing (DLP)	LED-based digital light processing (DLP)	LED-based digital light processing (DLP)	Digital light processing (DLP)
Printing orientation	0 degree	0 degree	0 degree	0 degree	0 degree
Cleaning protocol	Immersed in isopropyl alcohol 99.9% liquid (Saudi Pharmaceutical Industries, Riyadh, Saudi Arabia), then dried with air	Immersed in isopropyl alcohol 99.9% liquid (Saudi Pharmaceutical Industries, Riyadh, Saudi Arabia), then dried with air	Immersed in isopropyl alcohol 99.9% liquid (Saudi Pharmaceutical Industries, Riyadh, Saudi Arabia), then dried with air	Immersed in isopropyl alcohol 99.9% liquid (Saudi Pharmaceutical Industries, Riyadh, Saudi Arabia), then dried with air	Immersed in isopropyl alcohol 99.9% liquid (Saudi Pharmaceutical Industries, Riyadh, Saudi Arabia), then dried with air
Printer	Asiga Max, technology: DLP wavelength 385 nm (ASIGA, Sydney, Australia)	Asiga Max, technology: DLP wavelength 385 nm (ASIGA, Sydney, Australia)	Asiga Max, technology: DLP wavelength 385 nm (ASIGA, Sydney, Australia)	Asiga Max, technology: DLP wavelength 385 nm (ASIGA, Sydney, Australia)	NextDent 5100 printer, technology: DLP wavelength 405 nm (NextDent 5100 printer, Nextdent B.V., Soesterberg, The Netherlands)
Curing process, time, and temperature	Asiga^®^ Flash Cure Box (Asiga, Sydney, Australia) for 30 min (15 min for each surface) at 60 °C curing temperature	Asiga^®^ Flash Cure Box (Asiga, Sydney, Australia) for 30 min (15 min for each surface) at 60 °C curing temperature	Asiga^®^ Flash Cure Box (Asiga, Sydney, Australia) for 30 min (15 min for each surface) at 60 °C curing temperature	Asiga^®^ Flash Cure Box (Asiga, Sydney, Australia) for 30 min (15 min for each surface) at 60 °C curing temperature	LC-3DPrint Box (3D Systems Corporation, Rock Hill, SC, USA) for 30 min (15 min for each surface) at 60 °C curing temperature
Storage condition	After printing, stored at room temperature away from bright light	After printing, stored at room temperature away from bright light	After printing, stored at room temperature away from bright light	After printing, stored at room temperature away from bright light	After printing, stored at room temperature away from bright light

**Table 2:** The baseline and post-scaling roughness values (Sa, µm) following the scaling (mean ± SD). Different letters indicate significant differences using paired *t*-test.

	Plastic Scaler		Hand Scaler		Ultra-Sonic Scaler	
Crowntec	0.18 ± 0.01 ^a^	0.20 ± 0.02 ^b^	0.22 ± 0.01 ^a^	0.30 ± 0.15 ^a^	0.14 ± 0.01 ^a^	0.29 ± 0.10 ^b^
Varseosmile	0.15 ± 0.02 ^a^	0.19 ± 0.03 ^b^	0.20 ± 0.02 ^a^	0.27 ± 0.16 ^a^	0.16 ± 0.02 ^a^	0.32 ± 0.13 ^b^
Freeprint	0.17 ± 0.02 ^a^	0.24 ± 0.04 ^b^	0.18 ± 0.03 ^a^	0.24 ± 0.17 ^a^	0.18 ± 0.02 ^a^	0.33 ± 0.07 ^b^
Ezprint	0.22 ± 0.02 ^a^	0.27 ± 0.07 ^b^	0.19 ± 0.04 ^a^	0.24 ± 0.08 ^a^	0.16 ± 0.02 ^a^	0.32 ± 0.06 ^b^
C&B Nextdent	0.19 ± 0.03 ^a^	0.19 ± 0.03 ^a^	0.22 ± 0.02 ^a^	0.29 ± 0.15 ^a^	0.15 ± 0.03 ^a^	0.33 ± 0.07 ^b^

## Data Availability

The original contributions presented in this study are included in the article. Further inquiries can be directed to the corresponding author.
